# The p12 Domain Is Unstructured in a Murine Leukemia Virus p12-CA^N^ Gag Construct

**DOI:** 10.1371/journal.pone.0001902

**Published:** 2008-04-02

**Authors:** Sampson K. Kyere, Prem Raj B. Joseph, Michael F. Summers

**Affiliations:** Howard Hughes Medical Institute, Department of Chemistry and Biochemistry, University of Maryland, Baltimore, Maryland, United States of America; National Cancer Institute, United States of America

## Abstract

The Gag polyproteins of gammaretroviruses contain a conserved p12 domain between MA and CA that plays critical roles in virus assembly, reverse transcription and nuclear integration. Here we show using nuclear magnetic resonance, that p12 is unstructured in a Moloney murine leukemia virus (MMLV) Gag fragment that includes the N-terminal domain of CA (p12-CA^N^). Furthermore, no long range interactions were observed between the domains, as has been previously predicted. Flexibility appears to be a common feature of Gag “late” domains required for virus release during budding. Residues near the N-terminus of CA^N^ that form a β-hairpin in the mature CA protein are unfolded in p12-CA^N^, consistent with proposals that hairpin formation helps trigger capsid assembly.

## Introduction

All retroviruses encode a polyprotein called Gag that serves as the major structural protein of the virus and is capable of assembling into virus-like particles in the absence of any other viral constituent. Gag proteins contain three major domains: an N-terminal matrix (MA) domain that regulates intracellular trafficking and membrane targeting, a capsid (CA) domain (that consists of N- and C-terminal subdomains) that promotes virus assembly and forms the capsid shell of the viral core during proteolytic maturation, and a nucleocapsid (NC) domain that is responsible for genome selection and encapsidation. Atomic level structures have been determined for several MA, CA and NC domains, and in general, the domain structures do not vary significantly among different genera of retroviruses [1], [2].

Gag proteins contain additional polypeptide elements and spacers that are often critical for proper virus assembly. In particular, short, proline-rich segments have been identified that facilitate viral budding at late stages of the viral lifecycle, and mutations within these segments typically give rise to elongated or partially formed virus-like buds that are not released from the plasma membrane [3]–[11]. These “late domains” are typically located in disparate regions of the Gag proteins of different retroviruses [9]. The late budding activity of the human immunodeficiency virus type-1 (HIV-1) is mediated by a conserved PT/SAP segment that resides within the C-terminal p6 region of Gag [3], [6]. In contrast, the late budding activity of the gamma-retroviruses is mediated by a conserved PPPY motif found within an 84 residue polypeptide called p12, which is located between the N-terminal MA and CA domains [12]. Disruption of the Moloney murine leukemia virus (MMLV) PPPY element gives rise to budding defects similar to those observed for HIV-1 PT/SAP mutants [12].

Proteolytic cleavage of the p12-CA junction is critical for viral infectivity and precedes cleavage of both the MA-p12 and CA-NC sites [13]. The N-terminal residues of mature N-tropic murine leukemia virus (N-MLV) CA (identical with MMLV CA except for five amino acid substitutions) adopt a β-hairpin that makes intermolecular CA-CA contacts upon hexamer formation [14]. It is likely that cleavage of the p12-CA junction is required for both hairpin formation and capsid assembly, as appears to be the case for HIV-1 capsid assembly [15], [16]. Cryo-electron microscopy (EM) images of immature MMLV virions revealed a low density zone of 25–60 Å between the MA and CA domains, suggesting that p12 is either unfolded or folded but highly mobile prior to proteolysis [17]. In addition to its role in virus assembly, the mature p12 peptide facilitates reverse transcription and the delivery of the pre-integration complex (PIC) to the nucleus for integration of the viral DNA into the host genome during the early phase of infection [18]–[23]. To further understand the structure and diverse roles of p12 as well as the cooperative nature of p12 and CA, we have characterized the dynamic properties and solution structure of a recombinant construct composed of p12 and the N-terminal domain of CA (CA^N^).

## Methods

The pNCA MMLV proviral plasmid [24] was used to subclone MMLV p12CA^N^, with a C-terminal His-tag, into pET11a (Novagen) using NdeI and BamHI restriction sites, and subsequently transformed into BL21 codon plus RP (DE3) cells (Stratagene). Cells were grown in LB or M9 minimal media supplemented with 99.9 % enriched ^15^N-ammonium chloride and/or 99.8 % enriched ^13^C-glucose as the sole nitrogen and/or carbon sources. Protein expression was induced in shake flasks with 1 mM IPTG. The cells were harvested and lysed with a microfluidizer (Micorfluidics), clarified by centrifugation, and the target protein was purified to homogeneity using cobalt affinity (Talon), cation exchange and size exclusion chromatographies (Amersham). Fractions containing pure protein were concentrated using Centripreps (Amicon, MWCO = 3,500 Da) and Centricons (Amicon, MWCO = 3,500 Da). The mass of p12CA^N^ was confirmed by mass spectroscopy.

NMR data were collected at 35°C with a Bruker AVANCE 600 MHz spectrometer equipped with a cryoprobe using samples of 0.8–1.0 mM p12CA^N^ in buffer containing 50 mM sodium phosphate pH 5.5, 100 mM NaCl, 5 mM DTT and 10% D_2_O. Backbone assignments were obtained using 2D ^1^H,^15^N-HSQC, 3D ^15^N-edited TOCSY, and 3D HNCA and HN(CO)CA experiments [25]-[29]. 3D ^15^N-edited NOESY-HSQC, 4D ^13^C,^15^N-edited NOESY and 4D ^13^C,^13^C-edited NOESY experiments were used for side chain assignments and to identify long range intra- and interdomain [^1^H,^1^H] NOEs [30].

To gain insight into the dynamical properties of MMLV p12, {^1^H}–^15^N steady-state heteronuclear NOE (XNOE) data were obtained for the backbone ^15^N nuclei [31]. The XNOE experiment consists of a Reference and NOE experiment collected in an interleaved mode. The reference experiment contained a recovery delay of 8 sec and the NOE experiment applied proton saturation during the last 3 sec of the 8 sec recovery delay [31]. The XNOE value for a given residue is derived from the intensity ratio (I/I_0_) of its ^15^N/^1^H correlation peak in the presence of 3 sec proton saturation (I) and in the absence of proton saturation (I_0_). Peak intensities and the XNOE value were calculated using the HetNOE Analysis module in NMRVIEW. Errors were estimated from the baseline noise in the two spectra. All NMR data were processed using NMRPipe [32] and analyzed using NMRView [33]. Backbone chemical shifts were deposited in the Biological Magnetic Resonance Bank; Accession number 15672.

## Results and Discussion

**Figure 1:**
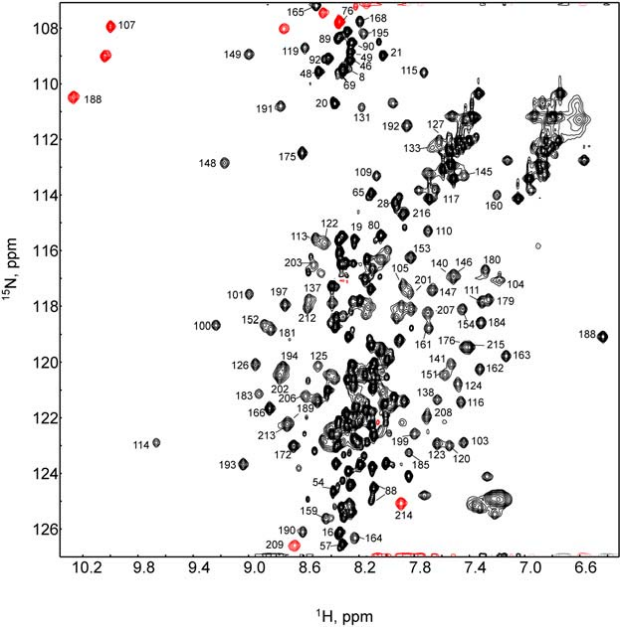
^1^H-^15^N correlation (HSQC) spectrum obtained for MMLV p12CA^N^. Assignments are shown for signals in less-crowded regions of the spectrum. Red peaks represent signals folded in the ^15^N dimension.

Initial examination of the 2D ^1^H,^15^N-HSQC spectra for p12CA^N^ revealed a large subset of signals with poor chemical shift dispersion in the proton dimension, suggestive of a largely unstructured domain within the protein (Figure 1). Backbone resonance assignments established that these signals corresponded to residues in the p12 domain. The high degree of spectra overlap made backbone assignments difficult for this domain, yielding 60 % of the resonances assigned for p12. However, backbone assignments were made for 95 % of the non-proline residues of CA^N^.

**Figure 2:**
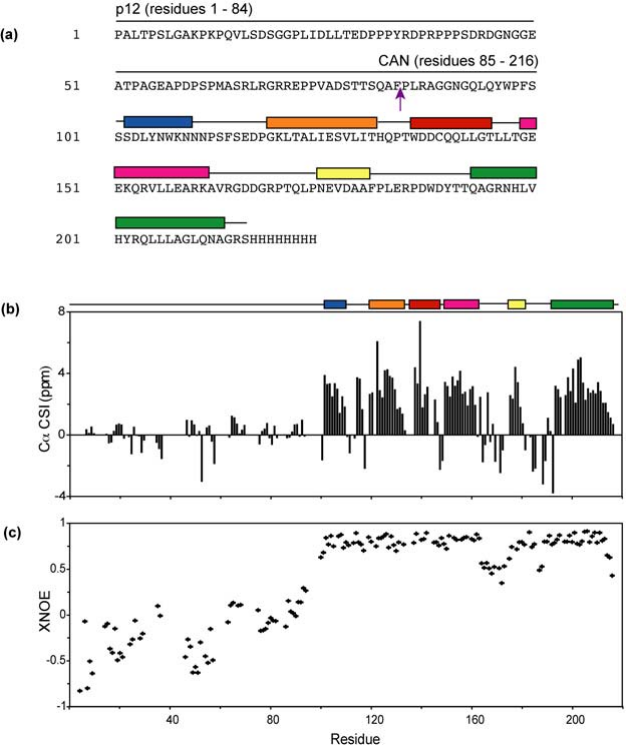
NMR chemical shift and relaxation data that identify regions of structure and mobility in p12CA^N^. (A) Amino acid sequence of p12CA^N^ (arrow denotes proteolytic cleavage site). Residues of CA^N^ that adopt α-helical conformations in the N-MLV CA^N^ crystal structure are denoted by colored rectangles. (B) NMR chemical shift indices for the backbone Cα atoms of p12CA^N^. Positive values denote helical regions, negative values denote regions of β-structure, and stretches of residues with near-zero values denote random coil conformations. For comparison, α-helical segments observed in the N-MLV CA^N^ crystal structure are aligned at the top of the panel. (C) ^15^N{^1^H} heteronuclear NOE (XNOE) data obtained for p12CA^N^. Values near 1.0 reflect reduced molecular motion, and smaller or negative values reflect motion on a fast (ps-ns) timescale.

Backbone Cα NMR chemical shift indices (CSI, the difference between the observed chemical shift and shifts observed for random coil structures) provide information on local secondary structure [34], [35], with downfield shifts (positive deviations) reflecting α-helical conformations and upfield shifts reflecting extended (β-strand) conformations. Zero or near zero CSIs were observed for all assigned residues of the p12 domain of p12CA^N^, indicating that the p12 domain is unstructured, Figure 2. In contrast, most residues of the CA^N^ domain of p12CA^N^ exhibit positive deviations (Figure 2), in agreement with the crystal structure of MMLV CA^N^ hexamer [14].

Atomic level structures have been determined for the mature CA^N^ domains of several retroviruses, and in all cases, the first ∼15 residues form a β-hairpin stabilized by a salt bridge between Pro 1 and the carboxyl group of an Asp residue subsequent to proteolytic induced maturation [14], [15], [36]–[39]. These residues are unstructured in an HIV-1 MA-CA Gag construct, indicating that β-hairpin formation occurs subsequent to proteolytic cleavage of the MA-CA junction [40]. Near-zero CSIs observed here for assigned N-terminal residues of the CA^N^ domain of p12CA^N^ indicate that the β-hairpin observed in the X-ray structure of the mature MMLV CA^N^ domain is similarly unfolded in the immature p12CA^N^ construct.

To gain insight into the dynamical properties of N-MLV p12, {^1^H}–^15^N steady-state heteronuclear NOE (XNOE) data were obtained for the backbone ^15^N atom [31]. XNOEs provide information regarding high-frequency (psec-nsec) backbone motions and are therefore useful for identifying regions of the protein with high internal mobility. The maximum theoretical XNOE value of 0.86 reflects highly restricted internal motion, whereas values smaller than ∼0.7 are indicative of substantial internal motion [31]. XNOE values were measured for 44 residues of p12 and 106 of CA^N^ that were well resolved in the 2D ^1^H,^15^N-HSQC spectra [31]. The residues of p12 exhibited primarily negative or near zero XNOEs, indicative of a high degree of internal flexibility and consistent with the random coil CSI analysis. The high degree of mobility extends through the first 16 residues of the CA^N^ domain, confirming that the β-hairpin is unfolded in the immature p12CA^N^ construct. In contrast, residues within the α-helices of the CA^N^ domain exhibit relatively large XNOE values, consistent with a regularly folded tertiary structure.

Multi-dimensional ^1^H-^1^H NOE data (from 3D ^15^N-edited NOESY-HSQC, 4D ^13^C,^15^N-edited NOESY and 4D ^13^C,^13^C-edited NOESY experiments; data not shown) were also obtained to probe for intra- and potential inter-domain contacts. Except for the N-terminal residues of the CA^N^ domain, which did not exhibit long-range interactions, the intra-CA^N^ NOEs observed in all the NOE data were fully consistent with the X-ray structure of the mature CA^N^ hexamer. No long-range intra- or inter-domain interactions were observed for residues of p12.

The combined CSI, XNOE and ^1^H-^1^H NOE NMR data indicate that the p12 domain of MMLV p12CA^N^ is conformationally labile. The folded regions of the HIV-1 MA and CA domains are also separated by a stretch of flexible residues (∼20 in the MA-CA^N^ NMR structure) [40], and flexibility between these domains may be important for allowing the CA-CA interactions to adjust during virus assembly and maturation. Flexibility in the p12 domain may also be required for interactions with host cell proteins involved in late assembly processes, including viral release. The PTAP element of HIV-1 Gag, which interacts directly with TSG101 [9], [41]–[45] (a component of the cellular protein sorting machinery), is also highly flexible in solution [9]. The inherent flexibility allows p6 to bind to a cleft on TSG101 UEV domain via an induced-fit mechanism [46], [47].

In summary, the MMLV p12 domain of p12CA^N^ exhibits structural/dynamical properties similar to those observed for HIV-1 p6, despite that fact that p12 is twice as large as p6, is located in a very different region of Gag, and has additional functions during the early phase of viral replication.
